# Supplementary material for: Genetic and Clinical Characteristics of Korean Chronic Lymphocytic Leukemia Patients with High Frequencies of MYD88 Mutations
Source: Int J Mol Sci. 2023 Feb 6;24(4):3177. doi: 10.3390/ijms24043177 (PMC9959581; doi:10.3390/ijms24043177)
Supplement: Supplementary file 1 [file ijms-24-03177-s001.zip › IJMS_CLL_Supplemental_Material.pdf]

Supplemental Materials

Supplemental Tables

Supplemental Table S1. Gene targets of SM CLL/lymphoma panel

Transcription regulators	*ARID1B, ARID2, BCORL1, CREBBP, KMT2A, KMT2D, KDM6A, TET2*
TLR signaling pathway	*MYD88*
NF-κB signaling pathway	*BIRC3, CARD11, MAP2K1, TNFAIP3, TRAF3*
BCR signaling pathway	*CD28, CD79A, CD79B, TNFRSF14*
MAPK signaling pathway	*BRAF, KRAS, NF1, NRAS*
DNA damage and repair	*ATM, TP53*
RNA splicing and processing	*SF3B1*
Others	*APC, CCND1, CCND2, CHD8, DIS3, DNMT3A, EZH2, FOXO1, GNA13, ID3, IDH2, NOTCH1, NOTCH2, RHOA, STAT3, STAT5B, TCF3, ZAP70*

Abbreviations: TLR, Toll-like receptor; MAPK, mitogen-activated protein kinase; SM CLL, St. Mary's chronic lymphocytic leukemia

Supplemental Table S2. List of detected mutations

See the file "CLL Supplementary Table 2.xlsx"

Supplemental Table S3. Univariate and multivariate Cox proportional hazards regression analyses of genetic factors for time to treatment

Variables	Univariate analysis			Multivariate analysis		
	HR	(95% CI)	*p*	HR	(95% CI)	*p*
Somatic hypermutation	0.40	(0.19-0.84)	0.016	0.57	(0.23-1.42)	0.226
Isolated del(13q)	1.93	(0.74-5.01)	0.178			
Trisomy 12	1.67	(0.62-4.50)	0.313			
Complex karyotype	1.74	(0.70-4.33)	0.237			
ATM abnormality	1.86	(0.72-4.80)	0.203			
TP53 abnormality	2.95	(1.22-7.12)	0.016	2.86	(1.17-7.00)	0.021
NOTCH1 mutation	4.51	(1.87-10.91)	<0.001	4.99	(1.49-16.69)	0.009
MYD88 mutation	0.68	(0.34-1.39)	0.292			

Abbreviations: HR, hazard ratio; CI, confidence interval

Supplemental Table S4. Univariate and multivariate Cox proportional hazards regression analyses of genetic factors for overall survival

Variables	Univariate analysis			Multivariate analysis		
	HR	(95% CI)	*p*	HR	(95% CI)	*p*
Somatic hypermutation	0.16	(0.04-0.60)	0.006	0.50	(0.10-2.51)	0.403
Isolated del(13q)	1.39	(0.14-13.50)	0.774			
Trisomy 12	1.42	(0.17-11.64)	0.741			
Complex karyotype	1.45	(0.16-13.05)	0.738			
ATM abnormality	1.69	(0.20-14.15)	0.630			
TP53 abnormality	7.26	(1.73-30.58)	0.007	7.07	(1.56-32.1)	0.001
NOTCH1 mutation	7.21	(1.45-35.88)	0.016	3.47	(0.44-27.6)	0.239
MYD88 mutation	0.56	(0.12-2.64)	0.463			

Abbreviations: HR, hazard ratio; CI, confidence interval
